# Gallstone Ileus as an Uncommon Etiology of Intestinal Obstruction: A Case Report

**DOI:** 10.7759/cureus.87780

**Published:** 2025-07-12

**Authors:** Salvador Pelayo-González, Carlos Iskyam Zaldo-Arredondo, Alejandro Mateos-Bear, Francisco J Ruiz-Lara, Ivana S Vázquez-Villanueva, Diego A Saldaña-Rentería, Quitzia L Torres-Salazar

**Affiliations:** 1 General Surgery, Hospital General de León, León, MEX; 2 Biomedical Sciences, Universidad Juárez del Estado de Durango, Durango, MEX

**Keywords:** bilioenteric fistula, enterolithotomy, gallstone ileus, rigler’s triad, small bowel obstruction

## Abstract

Although gallstones are commonly confined to the biliary system, in rare instances, they may migrate into the intestinal tract and cause obstruction, a scenario most often observed in older adults with a history of gallbladder pathology. We describe the case of a 46-year-old woman who developed small bowel obstruction secondary to a large impacted gallstone, despite having no prior biliary disease. Her clinical presentation included a 15-day history of abdominal pain, bilious vomiting, and diarrhea. Laboratory tests revealed leukocytosis and acute kidney injury. Contrast-enhanced computed tomography demonstrated pneumobilia, small bowel obstruction, and a radiopaque ectopic gallstone. Exploratory laparotomy confirmed a 4 × 3 × 4 cm gallstone impacted 30 cm proximal to the ileocecal valve, which was successfully extracted via longitudinal enterotomy. The postoperative recovery was uneventful. This case illustrates the diagnostic and surgical challenges posed by gallstone ileus and emphasizes the importance of considering this condition even in patients without prior biliary pathology. Enterolithotomy proved to be a safe and effective treatment in this setting.

## Introduction

Gallstone ileus is an uncommon but significant cause of mechanical small bowel obstruction, accounting for 1-4% of all intestinal obstruction cases and up to 25% among elderly patients [1]. It occurs when a large gallstone enters the gastrointestinal tract through a bilioenteric fistula (most commonly a cholecystoduodenal fistula) and becomes impacted in the intestinal lumen, typically near the ileocecal valve (where the intestinal lumen gradually narrows, making it the most common site of impaction), causing acute mechanical obstruction [2].

This condition usually develops in the setting of chronic cholecystitis and prolonged inflammation, where large, immobile gallstones erode the gallbladder wall in contact with adjacent bowel loops. In some patients, this process occurs as a late complication of Mirizzi syndrome, a rare condition in which a gallstone compresses or erodes into the common bile duct and may progress to fistula formation [3].

Gallstone ileus poses a diagnostic challenge due to its nonspecific clinical presentation, which often includes abdominal pain, vomiting, and distension, symptoms that can mimic other etiologies of intestinal obstruction. Although Rigler's triad (pneumobilia, ectopic gallstone, and intestinal obstruction) is considered pathognomonic, it is not universally present on imaging [1]. The condition typically results from chronic inflammation of the gallbladder, leading to fistula formation, through which a preformed large stone directly migrates into the bowel. 

Prompt recognition of this entity and its radiological features (especially through computed tomography) is crucial for timely surgical management and to avoid complications such as bowel ischemia or perforation. We present the case of a 46-year-old woman with a history of extreme prematurity (born at 6 months and 13 days of gestation), who required prolonged mechanical ventilation and CPAP due to neonatal respiratory distress syndrome and later developed sequelae of cerebral palsy. At presentation, she was also malnourished. While rare, extreme prematurity is a recognized risk factor for neonatal cholestasis, particularly in those requiring intensive respiratory support and prolonged hospitalization. These factors, along with long-term neuromotor impairment and nutritional deficits, may contribute to hepatobiliary dysfunction and increase the risk of cholelithiasis later in life. This case highlights the diagnostic complexity and the importance of timely recognition and surgical management of this rare condition. This case has been reported in accordance with the SCARE (Surgical CAse REport) 2025 criteria [4].

## Case presentation

A 46-year-old woman with a history of extreme prematurity (born at 6 months and 13 days of gestation), neonatal respiratory distress syndrome treated with prolonged CPAP, mild sequelae of cerebral palsy, and chronic malnutrition presented to the emergency department with a 15-day history of progressive gastrointestinal symptoms. She reported vomiting of gastric contents occurring four times daily, associated with watery diarrhea three times per day and intermittent abdominal pain. An initial outpatient diagnosis of infectious gastroenteritis was made based solely on clinical findings; no laboratory or imaging studies were performed prior to initiating treatment. She received intramuscular ceftriaxone, oral diphenidol, metoclopramide, and loperamide, with no clinical improvement.

Furthermore, the patient’s relatively young age (falling outside the typical age group for gallstone ileus, which predominantly affects the elderly) likely contributed to the initial misdiagnosis, as this condition was not initially considered within the differential diagnoses.

Upon presentation to the emergency department, the patient appeared uncomfortable, with dry oral mucosa and a distended, rigid, and tender abdomen, particularly in the epigastric and hypogastric regions. Bowel sounds were diminished, and digital rectal examination revealed the absence of stool in the rectal ampulla. No peripheral edema or signs of deep vein thrombosis were observed. Her medical history was notable for recently diagnosed hypertension, managed with telmisartan. She had no history of prior abdominal surgeries.

Initial laboratory evaluation revealed leukocytosis (13.5 ×10⁹/L), elevated urea (180 mg/dL), creatinine (2.56 mg/dL), total bilirubin (3.2 mg/dL), and hypercholesterolemia (380 mg/dL) (Table 1).

**Table 1:** Laboratory parameters on admission. Hb: Hemoglobin (g/dL); Hct: hematocrit (%); WBC: white blood cell count (×10⁹/L); Na: sodium (mEq/L); K: potassium (mEq/L); AST (SGOT): aspartate aminotransferase (U/L); ALT (SGPT): alanine aminotransferase (U/L); TBil: total bilirubin (mg/dL); DBil: direct bilirubin (mg/dL); IBil: indirect bilirubin (mg/dL); HDL: high-density lipoprotein (mg/dL); CRP: C-reactive protein (mg/L); PT: prothrombin Time (s); aPTT: activated partial thromboplastin time (s).

Parameter	Patient Value	Reference Range
Hb	13.6	12-16 g/dL
Hct	40.7	36-46%
WBC	13.5	4.0-10.0 x10⁹/L
Glucose	103	70-100 mg/dL
Urea	180	15-40 mg/dL
Creatinine	2.56	0.6-1.2 mg/dL
Na	137	135-145 mEq/L
K	3.8	3.5-5.0 mEq/L
SGOT	85	10-40 U/L
ALT (SGPT)	31	10-40 U/L
TBil	3.2	0.2-1.2 mg/dL
DBil	1.8	<0.3 mg/dL
IBil	1.4	<0.9 mg/dL
Total cholesterol	380	<200 mg/dL
HDL cholesterol	28	>40 mg/dL
Triglycerides	269	<150 mg/dL
CRP	5.6	<3.0 mg/L
PT	14	11-13.5 s
aPTT	21	25-35 s

**Figure 1:**
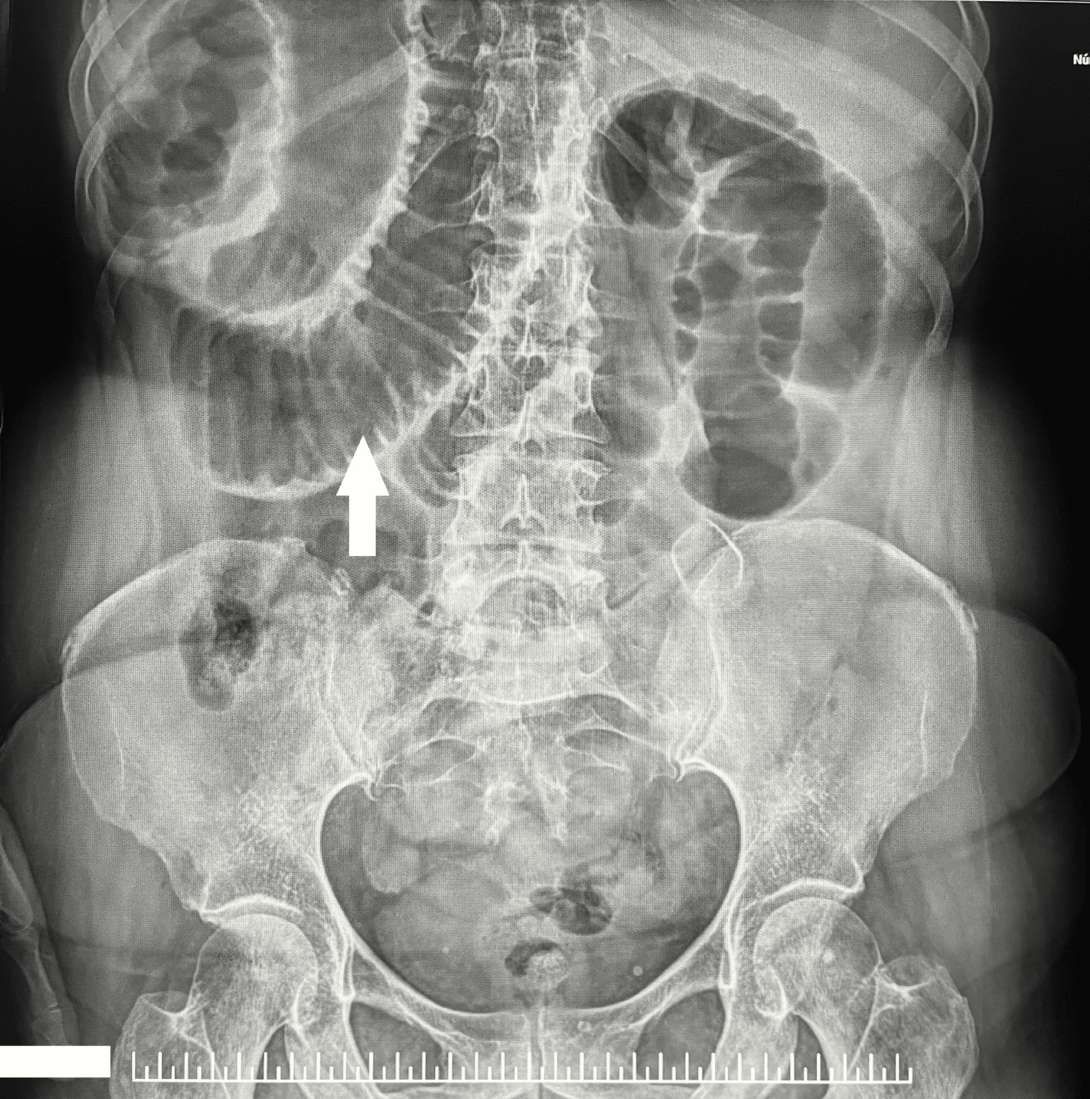
Female patient presenting with marked abdominal distension, with imaging revealing "stacked coin" appearance, suggestive of dilated bowel loops.

**Figure 2:**
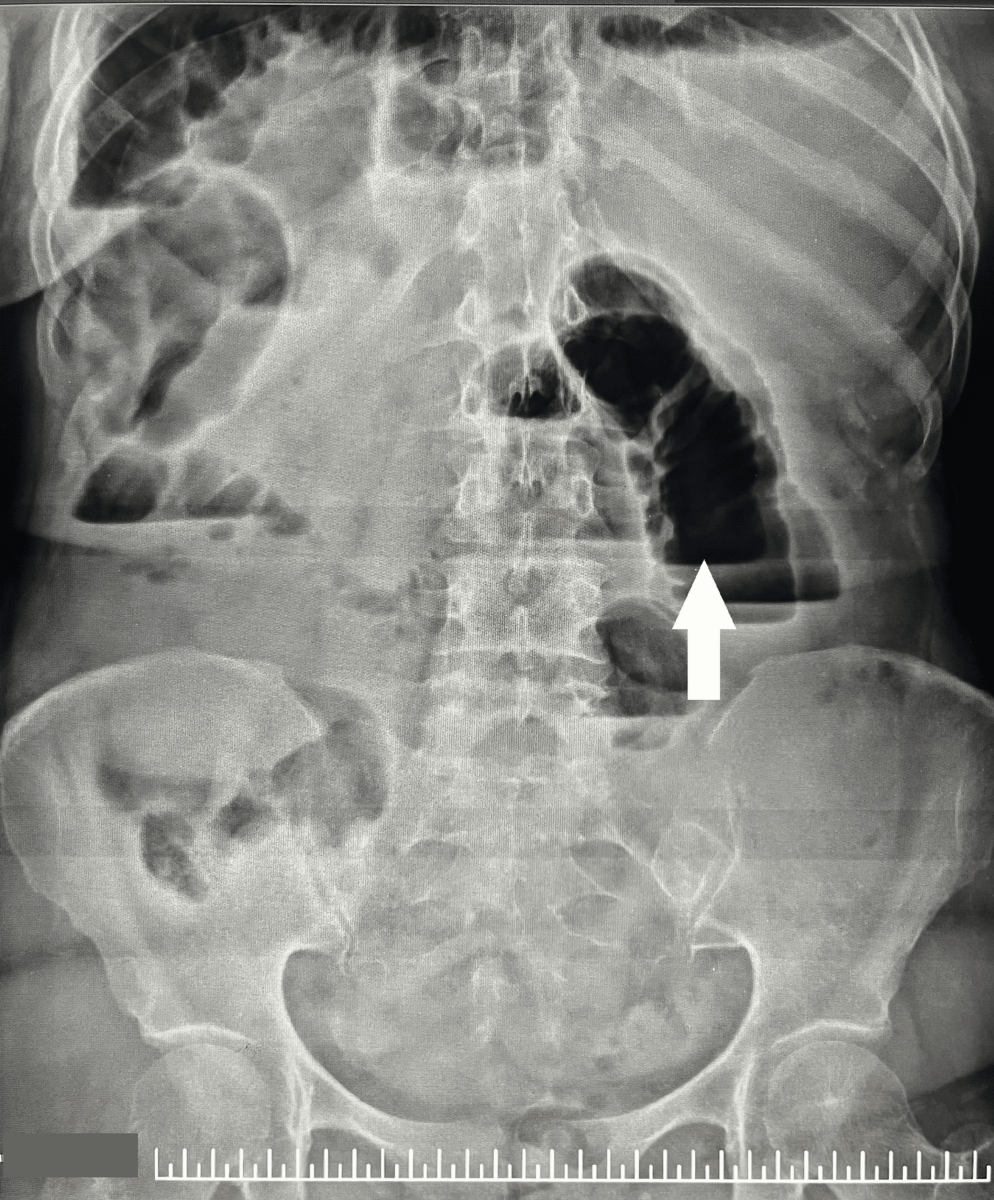
Presence of air-fluid levels.

Liver transaminases were mildly elevated, and inflammatory markers were raised. Abdominal radiography showed dilated bowel loops with air-fluid levels (Figures 1-2).

**Figure 3:**
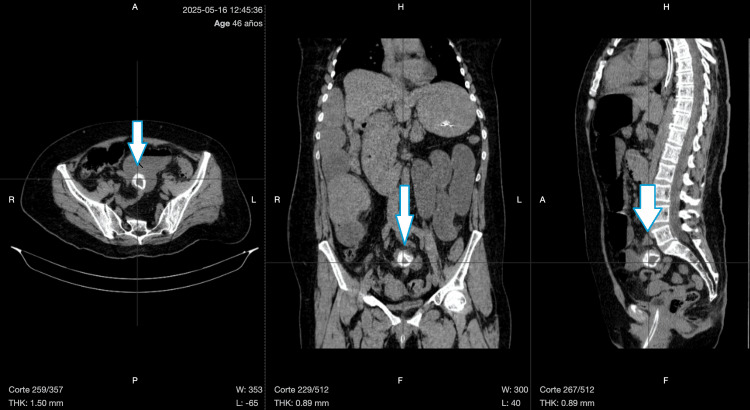
Contrast-enhanced abdominal computed tomography scan in axial (left), coronal (middle), and sagittal (right) planes. The blue arrows indicate a well-defined, hyperdense intraluminal gallstone located in the distal ileum, consistent with mechanical bowel obstruction.

A contrast-enhanced computed tomography (CT) scan of the abdomen revealed features consistent with gallstone ileus, including pneumobilia, a radiopaque intraluminal mass in the distal ileum, and proximal small bowel dilatation consistent with Rigler's triad (Figure 3).

**Figure 4:**
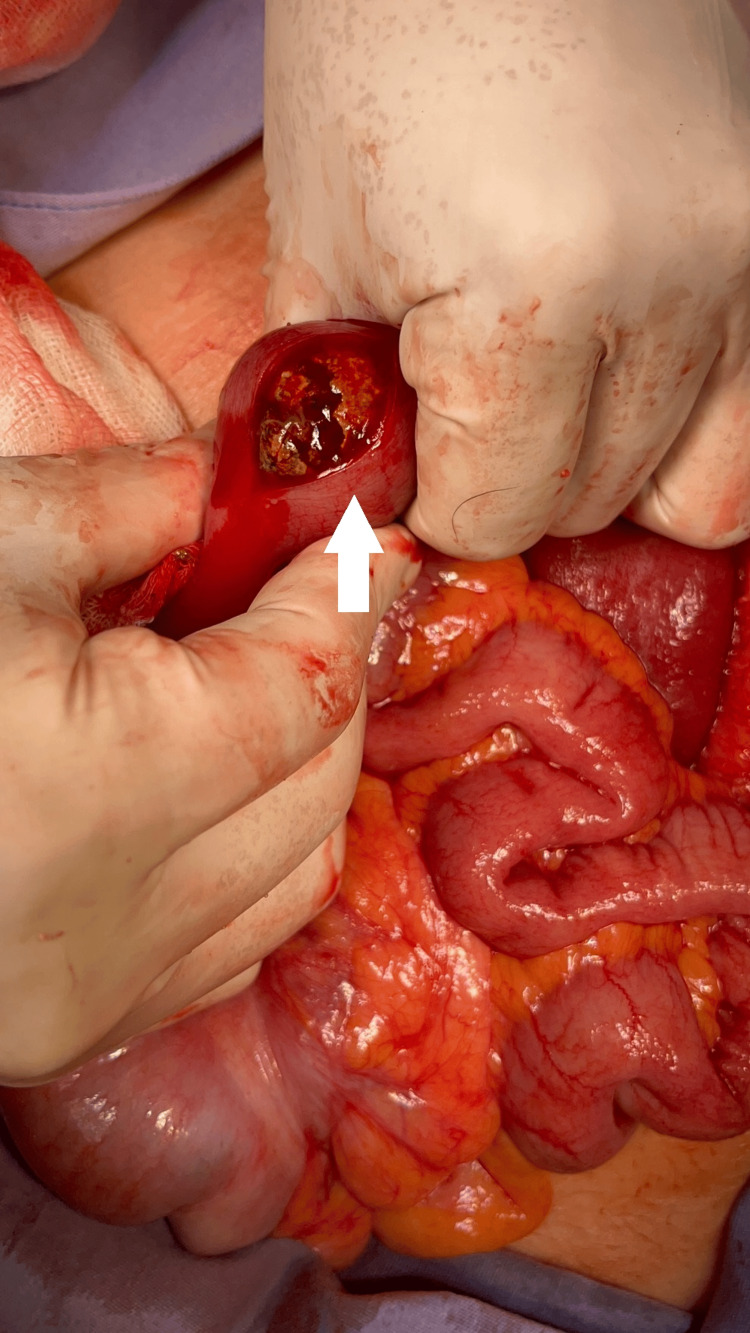
Gallstone located 30 cm from the ileocecal valve and 250 cm from the Treitz ligament. Longitudinal enterotomy with extraction of the gallstone

**Figure 5:**
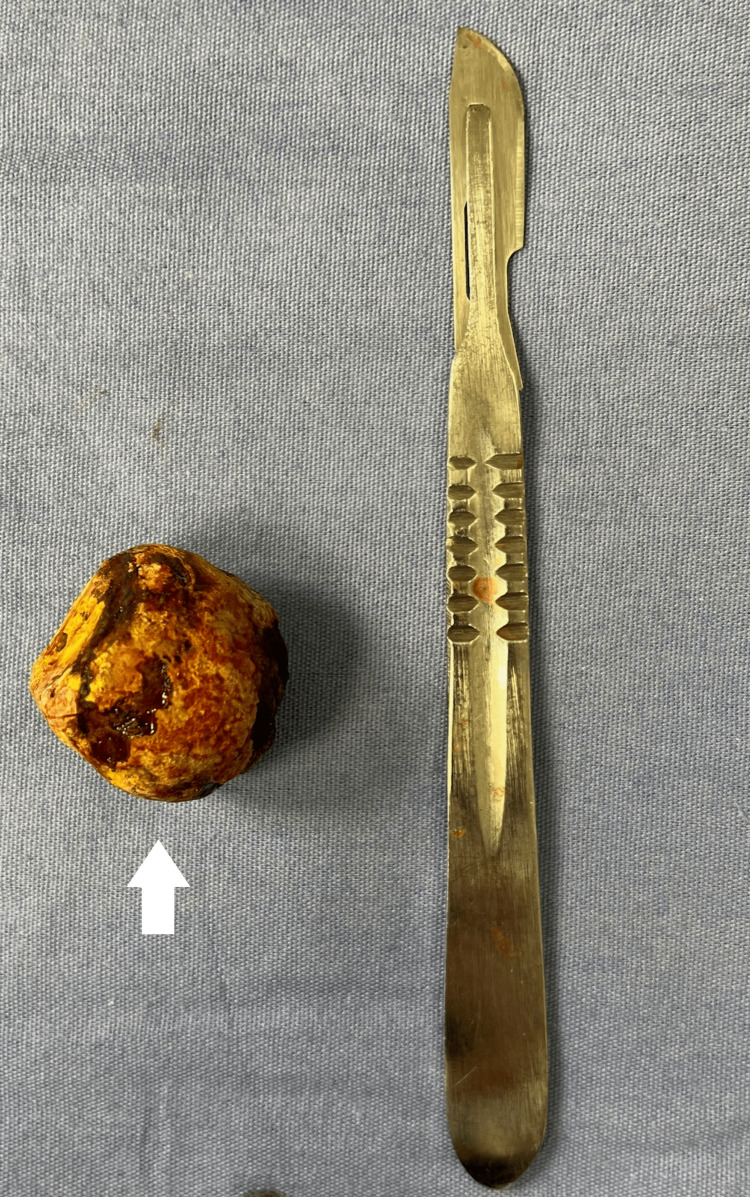
Retrieved gallstone measuring 4 × 3 × 4 cm

Exploratory laparotomy identified a single large gallstone impacted 30 cm proximal to the ileocecal valve and approximately 250 cm distal to the ligament of Treitz. A longitudinal enterotomy was performed with successful extraction of the 4×3×4 cm stone. To minimize the risk of postoperative stenosis, the enterotomy was closed transversely using the Heineke-Mikulicz technique in two layers (Figures 4-5).

Hepatic bed exploration revealed a cholecystoduodenal fistula without evidence of active leakage or perforation. The fistula was not dismantled; instead, a Penrose drain was placed in proximity, and the surgical site was closed in layers. The postoperative course was uneventful; liver function tests normalized before discharge, which occurred on postoperative Day 5 without complications.

## Discussion

Gallstone ileus is a rare but potentially life-threatening cause of mechanical bowel obstruction, representing less than 0.5% of all small bowel obstruction (SBO) cases and occurring in only 0.3% to 0.5% of patients with cholelithiasis. It predominantly occurs in elderly patients with multiple comorbidities, making diagnosis and treatment particularly challenging [1,5]. In this context, our case is exceptional, as it involves a relatively young patient with no significant prior hepatobiliary history, aside from her remote history of extreme prematurity and chronic malnutrition. This deviation from the typical demographic profile contributed to diagnostic delay and underscores the importance of maintaining a high index of suspicion, even in non-geriatric patients.

Pathophysiologically, gallstone ileus develops when chronic inflammation of the gallbladder leads to the formation of a bilioenteric fistula (most commonly a cholecystoduodenal fistula), allowing a gallstone to migrate into the gastrointestinal tract [6,7]. In some cases, this process occurs as part of Mirizzi syndrome type V, which involves external compression or erosion of the common bile duct by an impacted gallstone, progressing to fistula formation (type Va: without cholecystoenteric fistula; type Vb: with cholecystoenteric fistula) [3]. In our case, the stone caused a mechanical obstruction at the ileum, consistent with the most frequent impaction site due to its narrow lumen and lower peristaltic activity [1]. Stones larger than 2.5 cm are usually implicated in obstruction, with rare instances of stones exceeding 4 cm, such as the one presented by Matli et al., contributing to prolonged or severe obstructive symptoms and increased risk of ischemia [6].

Diagnosing gallstone ileus remains a challenge. Clinical features are often nonspecific and may mimic other causes of acute abdomen, including neoplastic obstruction or volvulus. The classic Rigler’s triad (pneumobilia, intestinal obstruction, and ectopic gallstone) can guide diagnosis, but is observed in fewer than 50% of cases on plain radiography [6]. As shown in multiple case reports, including our own and those by García-Quijada et al. [8] and Valencia-Martínez et al. [5], the use of contrast-enhanced abdominal CT significantly improves diagnostic yield, achieving a sensitivity up to 96.3% for bowel obstruction and up to 88.9% for pneumobilia [6]. CT also aids in identifying additional stones, signs of bowel ischemia, and the presence of bilioenteric fistulas.

In terms of management, enterolithotomy alone is widely accepted as the preferred treatment in elderly or high-risk patients due to its lower mortality rate (approximately 11.7%) compared to one-stage procedures, which include cholecystectomy and fistula repair (mortality up to 16.9%) [7,6]. As demonstrated in our case and supported by García-Quijada et al., the enterolithotomy approach effectively resolves the obstruction while minimizing perioperative stress and complications [8]. Although our patient was younger and without significant comorbidities, the intraoperative findings (inflammatory fluid, dilated bowel loops, and a single impacted intraluminal gallstone) and absence of residual stones or perforation supported the decision to avoid a one-stage procedure. A drainage tube was placed in the area of the fistulous bed, and follow-up was arranged to monitor for potential recurrence or biliary complications.

It is essential to inspect the entire gastrointestinal tract during surgery, as retained gallstones can result in persistent or recurrent obstruction. García-Quijada et al. reported that up to 16% of cases involve multiple stones, underscoring the importance of thorough palpation and inspection of the bowel during laparotomy [8]. Our surgical team adhered to this principle, ensuring the absence of additional calculi, thus reducing the risk of recurrence.

Although bowel ischemia and necrosis are relatively rare complications, they are more likely to occur in patients with delayed presentation or in cases of closed-loop obstruction, as in the double gallstone scenario reported by García-Quijada et al. Our patient showed no signs of ischemia or perforation, allowing for conservative resection avoidance [8]. However, segmental bowel resection may be necessary in 10-20% of cases, particularly when necrosis is present.

Endoscopic lithotripsy has been proposed as a less invasive option, particularly for cases of Bouveret syndrome or colonic gallstone ileus [6]. Nevertheless, this technique requires advanced endoscopic expertise and is limited to highly selected patients. It is not currently considered standard for most small bowel obstructions caused by gallstones.

From a prognostic standpoint, early identification and surgical management are pivotal. Mortality remains elevated, particularly in elderly and frail individuals, with reported rates ranging from 6% to 18% depending on the presence of sepsis, comorbid conditions, and timing of intervention [5,7]. In our case, although the patient had a 15-day delay in diagnosis, her young age, absence of significant comorbidities, and preserved physiological reserve likely contributed to avoiding complications such as bowel ischemia or sepsis. Timely CT diagnosis, fluid resuscitation, and prompt surgical intervention ultimately led to a favorable outcome, with full postoperative recovery and discharge without complications.

This case also underscores the diagnostic challenges posed by gallstone ileus, particularly when it occurs in younger patients outside the typical demographic profile for this condition. The initial misdiagnosis as infectious gastroenteritis and the resulting two-week delay in appropriate management highlight the importance of maintaining a high index of suspicion, even in non-elderly individuals.

## Conclusions

Gallstone ileus remains an infrequent but clinically significant cause of mechanical bowel obstruction. Although it most commonly affects elderly patients with multiple comorbidities, it can also present in younger individuals with few or atypical risk factors, as illustrated in this case. Such demographic variation may contribute to diagnostic delay. A high index of suspicion is essential, especially when imaging reveals signs suggestive of Rigler’s triad.

Contrast-enhanced CT plays a critical role in identifying the obstructing calculus and guiding timely surgical management. In this case, enterolithotomy alone proved to be an effective and safe approach. Intraoperative exploration of the entire bowel remains essential to prevent recurrence. This report reinforces the importance of individualized surgical decision-making and diagnostic vigilance, even in patients who fall outside the typical profile, to achieve favorable outcomes.
